# Establishment and Characterization of Canine Mammary Gland Carcinoma Cell Lines With Vasculogenic Mimicry Ability *in vitro* and *in vivo*

**DOI:** 10.3389/fvets.2020.583874

**Published:** 2020-10-27

**Authors:** Patrícia de Faria Lainetti, Andressa Brandi, Antonio Fernando Leis Filho, Maria Carolina Mangini Prado, Priscila Emiko Kobayashi, Renée Laufer-Amorim, Carlos Eduardo Fonseca-Alves

**Affiliations:** ^1^School of Veterinary Medicine and Animal Science, São Paulo State University—UNESP, Botucatu, Brazil; ^2^Institute of Health Sciences, Universidade Paulista-UNIP, Bauru, Brazil

**Keywords:** female dog, cell culture, mammary tumor, veterinary, oncology

## Abstract

Mammary tumors affect intact and elderly female dogs, and almost 50% of these cases are malignant. Cell culture offers a promising preclinical model to study this disease and creates the opportunity to deposit cell lines at a cell bank to allow greater assay reproducibility and more reliable validation of the results. Another important aspect is the possibility of establishing models and improving our understanding of tumor characteristics, such as vasculogenic mimicry. Because of the importance of cancer cell lines in preclinical models, the present study established and characterized primary cell lines from canine mammary gland tumors. Cell cultures were evaluated for morphology, phenotype, vasculogenic mimicry (VM), and tumorigenicity abilities. We collected 17 primary mammary carcinoma and three metastases and obtained satisfactory results from 10 samples. The cells were transplanted to a xenograft model. All cell lines exhibited a spindle-shaped or polygonal morphology and expressed concomitant pancytokeratin and cytokeratin 8/18. Four cell lines had vasculogenic mimicry ability *in vitro*, and two cell lines showed *in vivo* tumorigenicity and VM in the xenotransplanted tumor. Cellular characterization will help create a database to increase our knowledge of mammary carcinomas in dogs, including studies of tumor behavior and the identification of new therapeutic targets.

## Introduction

Mammary gland tumors frequently affect intact and older female dogs, and more than 50% of these cases are malignant (1, 2). Breast cancer (BC) is the main cause of mortality and the most common cancer type diagnosed in women (3). The occurrence of neoplasms in dogs is spontaneous and they share some similarities to BC in women, such as histological classification, molecular targets, and biological behavior. Therefore, canine mammary gland tumors are a natural model for human BC (4).

Female dogs with mammary carcinoma do not have the same therapeutic outcomes as women because chemotherapy treatment is not as effective in dogs, and it does not increase the patient's survival time or influence local disease control (5, 6). The gold standard treatment in dogs is radical mastectomy, and chemotherapy is performed depending on the tumor subtype, aggressiveness, or the presence of metastasis; the chosen protocols are based on human BC literature (1, 7, 8). Therefore, there is no standardized protocol for chemotherapy in female dogs affected by mammary gland tumors (6). There is also a lack of information on markers that predict antitumor responses, which are available for human BC treatment (9, 10). Therefore, canine cancer cell lines present a great opportunity for the evaluation of antitumor responses.

Cell lines are an alternative, experimental *in vitro* model of human BC and canine mammary gland tumors for the investigation of carcinogenesis processes, such as proliferation, apoptosis, and migration (11). Cell culture is an excellent preclinical model that is essential for the identification and evaluation of drug mechanisms of action, the identification of genes involved in carcinogenesis, such as oncogenes and tumor suppressors, the definition of the cell signaling pathways and their contribution to tumor pathogenesis, the discovery of new drugs, and the development process of antitumor drugs (12).

In canine and human patients with highly aggressive mammary neoplasms, the neoplastic cells may form vascular-like structures or channels, which are used to conduct plasma, red cells, and neoplastic cells during epithelial mesenchymal transition (13). The capacity of tumor cells to create non-endothelial vascular channels is called vasculogenic mimicry (VM) (14). The VM process occurs via the influence of cancer stem cells, which become endothelial-like cells and induce tumor neovascularization (13). The vessels formed during VM are composed of tumor and endothelial cells, and the newly generated vessels or channels are bonded to preexisting vessels (13, 14).

VM was studied as a mechanism of tumor nutrition and angiogenesis, and it may explain tumor metastasis (13–16). The presence of these vessels may be associated with a more aggressive tumor, a higher histopathological grade, shorter survival time, and a higher capacity of invasion and metastasis (14). The mechanisms involved in VM formation include the expression of markers related to epithelial–mesenchymal transition (EMT), stem cell properties, and hypoxia (14, 17, 18). EMT allows tumor cells to change their cytoskeleton in order to promote invasion and metastasis. During tubular formation, aggressive cells express EMT markers, acquire plasticity, and form vascular-like structures (19). During these processes, proteins such as E-cadherin, occludin-1, and α-catenin zone are downregulated while VE-cadherin, fibronectin, cadherin-2, and vimentin are upregulated (14, 17–19). Some receptors are also involved in the signaling of the VM pathway, such as ephrin type A receptor 2 (EphA2), focal adhesion kinase (FAK), phosphotidylinositol-3-kinase (PI3K), matrix metalloproteinase (MMP), Notch, and hypoxia-inducible factor 1-alpha (HIF1-α). These factors are involved in some way in modulating the formation of VM (14–18, 20, 21).

The development of therapies targeting VM may be relevant because this characteristic is closely linked to higher grade tumors, tumor aggressiveness, invasion rate, metastasis, and a worse prognosis (13, 18). Anti-angiogenic therapies focused on VM are not well-established due to the side effects of these drugs. Therefore, more studies on inhibitors of the signaling pathway for the formation of VM are needed (18). Some studies suggest the use of targeted drugs to inhibit FAK (22), EphA2 (21), MMP (23), and other receptors. VM was studied in inflammatory mammary carcinomas and other tumor subtypes in canines (24, 25). VM formation in humans has a poor clinical prognostic characteristic (14). Therefore, the present study established and characterized 10 cell lines from canine mammary gland tumors, including seven lines from primary tumors and three lines from metastases, according to immunophenotype, tumorigenicity, and the ability to form vascular-like structures *in vitro* and *in vivo*.

## Materials and Methods

### Animals and Experimental Design

This study was performed in accordance with the National and International Recommendations for the Care and Use of Animals (26). All procedures were performed after approval from the Ethics Committee on Animal Use (CEUA) of the Veterinary Teaching Hospital of São Paulo State University (CEUA/UNESP, #0208/2016 and #1267/2018).

### Reagents

All reagents used were of high purity and purchased from companies such as GE Healthcare (Uppsala, Sweden), Sigma-Aldrich (São Paulo, Brazil), Merck SA (São Paulo, Brazil), and other cited sources. Cell culture media included Mammary Epithelial Cell Growth Medium (MEGM^™^, Lonza Inc., Allendale, NJ, USA), fetal bovine serum (FBS; LGC Biotecnologia, Cotia, SP, Brazil), Dulbecco's phosphate-buffered saline (DPBS; Sigma-Aldrich, St. Louis, MO, USA), an antibiotic/antimycotic solution (Thermo Fisher Scientific, Waltham, MA, USA), and trypsin (0.25%, Gibco Thermo Fisher Scientific).

### Tumor Samples and Cell Isolation

Twenty samples of mammary gland tumors were collected at the Veterinary Medicine and Animal Science School, UNESP, between December 2016 and March 2017 for histopathological examination and cell culture. Some tumor samples were fixed in formalin and paraffin-embedded (FFPE); other samples were placed in MEGM^™^ media (Lonza Inc.) for immediate cell culture. Seventeen of the 20 samples were primary tumors and three samples were metastases. Cell lines were obtained using enzymatic dissociation, as previously described (27). Briefly, tumor fragments of ~1 cm^2^ were collected and dissociated using type IV collagenase (Sigma-Aldrich, St. Louis, MO) for 4 h at 37°C in a humidified atmosphere containing 5% CO_2_. Cells were separated using a 75-μm mesh filter, centrifuged, and washed with DPBS (Sigma-Aldrich, St. Louis, MO) to remove excess collagenase. Isolated cells were counted in a Neubauer chamber, and cell viability was evaluated using the Trypan blue technique. Plating was performed at a concentration of 1 × 10^4^ cells/ml in 25-ml culture bottles with filters.

Sample identification, histological classification, and the cell obtention technique are described in Table 1. Identification of the histological subtype and tumor grade were based on the international classification of mammary gland tumors (28).

**Table 1:** Mammary gland tumor information used to obtain neoplastic cells cultured *in vitro*.

**Identification**	**Breed**	**Age (years)**	Histologic classification^*^	Grade^*^	**Acquisition technique**	**Collagenase/ explant time**	**Actual culture passage**	Immunohistochemistry (IHC)^*^	**Tubular formation**
UNESP-CM1	Poodle	12	Solid carcinoma	Grade II	Collagenase	4 h	P10	HER2 overexpressing	Yes
UNESP-CM2	Teckel	15	Comedocarcinoma	Grade II	Collagenase	Overnight	–	–	–
UNESP-CM3	Poodle	13	Comedocarcinoma	Grade II	Collagenase	Overnight	–	–	–
UNESP-CM4	Teckel	13	Tubulopapillary	Grade II	Collagenase	4 h	P10	Triple-negative non-basal-like	No
UNESP-CM5	Teckel	10	Tubulopapillary	Grade II	Collagenase	4 h	P10	Triple-negative basal-like	No
UNESP-CM6	Pinscher	11	Carcinoma—mixed type	Grade I	Explant	15 days	–	–	–
UNESP-CM7	Poodle	15	Carcinoma—mixed type	Grade II	Explant	7 days	–	–	–
UNESP-CM8	Poodle	11	Comedocarcinoma	Grade II	Collagenase	4 h	–	–	–
UNESP-CM9	Mixed breed	12	Tubulopapillary	Grade II	Collagenase	4 h	P10	HER2 overexpressing	Yes
UNESP-CM10	Mixed breed	13	Carcinoma—mixed type	Grade I	Collagenase	4 h	–	–	–
UNESP-CM11	Mixed breed	13	Tubulopapillary	Grade I	Collagenase	3 h	P10	HER2 overexpressing	No
UNESP-CM12	German Shepherd	17	Carcinoma—mixed type	Grade II	Collagenase	4 h	–	–	–
UNESP-CM13	Beagle	17	Carcinoma—mixed type	Grade II	Collagenase	4 h	–	–	–
UNESP-CM14	Poodle	14	Carcinoma—mixed type	Grade I	Collagenase	4 h	–	–	–
UNESP-CM15	Akita	13	Carcinoma—mixed type	Grade II	Collagenase	4 h	–	–	–
UNESP-CM60	Teckel	14	Adenosquamous carcinoma	Grade II	Collagenase IV	3 h	P10	HER2 overexpressing	Yes
UNESP-CM61	Teckel	14	Comedocarcinoma	Grade III	Collagenase IV	3 h	P10	Triple-negative basal-like	No
UNESP-MM1	Poodle	12	UNESP-CM1 Bone metastasis	–	Collagenase	4 h	P10	Triple-negative basal-like	No
UNESP-MM3	Teckel	14	UNESP-CM61 Lymph node metastasis	–	Collagenase IV	3 h	P10	HER2 overexpressing	No
UNESP-MM4	Teckel	14	UNESP-CM60 Lymph node metastasis	–	Collagenase IV	3 h	P10	HER2 overexpressing	Yes

* *Neoplasms were classified and graded following Goldschmidt et al. (28). Immunohistochemical classifications were according to Nielsen et al. (29)*.

### Molecular Phenotype of Primary Tumors and Metastases

Immunohistochemistry (IHC) was performed on the FFPE samples. Sections (4-μm thickness) were placed onto positively charged slides (StarFrost, Braunschweig, Germany) and deparaffinized. Antigen retrieval was performed in citrate buffer (pH 6.0) in a pressure cooker (Pascal, Dako, Agilent Technologies, Santa Clara, CA, USA), and endogenous peroxidase was blocked using 8% hydrogen peroxide (Dinâmica Química Contemporânea, Indaiatuba, SP, Brazil) in methanol (Dinâmica Química Contemporânea) for 20 min. Non-specific protein binding was blocked using 8% skim milk for 60 min at room temperature. Primary antibodies against human epidermal growth factor receptor 2 (HER2), estrogen receptor alpha (ERα), progesterone receptor (PR), Ki-67, cytokeratin 5/6 (CK5/6), and epidermal growth factor receptor (EGFR) were diluted, and the samples were incubated according to Nguyen et al. (30) (Supplementary Table 1). Antibody detection was achieved using a polymer system (EnVision, Agilent Technologies). 3,3′-Diaminobenzidine (DAB) (EnVision, FLEX, High pH, Dako, Agilent Technologies) was used as the chromogen and tissue counterstaining was performed using Harris hematoxylin. Four human mammary carcinoma samples were used as positive controls for HER2: a negative sample, HER2 1+, HER2 2+, and HER2 3+. We evaluated our samples based on these staining patterns. For Ki67, CK5/6, and EGFR, we used adnexa glands from subcutaneous regions as a positive internal control. For ER and PR, we used canine uterine samples (31).

ERα and PR were considered positive when ≥10% of the nuclei were stained, CK5/6 and EGFRs were positive when cytoplasmic staining was ≥10%, and Ki-67 was positive when ≥33.3% of the cells had stained nuclei. For HER2 evaluation, more than 500 cells were scored randomly for labeling distribution and divided into four groups: score of 0, tumor cells did not show staining or ≤ 10% of the tumor cells showed weak staining; 1+, when ≥10% of the tumor cells had incomplete membrane staining; 2+, for moderate-to-strong staining in ≥10% of tumor cells; and 3+, when ≥10% of the tumor cells had complete and strong membrane staining. Scores of 0 and 1+ were considered negative and 2+ and 3+ were positive.

The molecular subtype was determined according to the previous human classification (10, 27). Briefly, the different molecular phenotypes of canine mammary carcinoma are classified as luminal A, luminal B, triple-negative, or HER2-overexpressing. Luminal A included tumors that are HER2-negative, ER- and/or PR-positive, and Ki67 ≤ 33%. Luminal B-type tumors are HER2-negative, ER- and/or PR-positive, and Ki67 ≥ 33%. Tumors that were HER2-, ER-, and PR-negative and EGFR- and/or CK5/6-positive were considered triple-negative basal-like tumors, and tumors that were HER2-, ER-, and PR-negative and EGFR- and/or CK5/6-negative were considered triple-negative non-basal-like tumors (29).

### Cell Expansion

Cell cultures were established from tumor fragments in MEGM^™^ containing 1% antibiotic/antimycotic solution and 10% FBS in a humid atmosphere containing 5% CO_2_ at 37°C. When the cells reached 80% confluence, the medium was discarded and the bottles were washed with sterile DPBS (pH 7.2) to eliminate residual FBS. For detachment of the cells from the bottle, trypsin 0.25% was added at 37°C, followed by a 5-min incubation period in a 5% CO_2_ humid atmosphere at 37°C. The cells were cultured until the 10th passage and used for cellular phenotyping, karyotype, morphology, and Western blotting.

### Contaminating Fibroblast Elimination

To eliminate fibroblasts from primary cultures, selective cell trypsinization using cold trypsin (4°C) was performed according to a previous study (32) at passage 5 (P5). Briefly, the cells were washed with DPBS at 4°C to avoid a direct thermal shock of cold trypsin. Two milliliters of cold trypsin (4°C) was used at room temperature (27°C) for 2 min. The supernatant was inactivated using complete medium containing 10% FBS in a 1:1 ratio, and trypsin was collected and discarded. The flasks were washed with DPBS buffer to remove residual trypsin. The cells were washed twice with a DPBS solution at room temperature and then washed once with DPBS buffer at 37°C. Trypsin (800 μl) at 37°C was added to the bottle and the cells were kept at 37°C in a humidified atmosphere containing 5% CO_2_ for 5 min. The remaining cells were detached via manual mechanical impact and the trypsin was inactivated with complete medium (containing 10% FBS) in a 1:1 ratio. The cells were centrifuged for 5 min at 1,200 rpm and resuspended in 5 ml of MEGM^™^ containing 10% FBS and a 1% antibiotic and antifungal solution.

### Cell Karyotype

Karyotype analysis was performed according to Moorhead et al. (33). Cells at P10 were cultured initially in MEGM^™^ medium supplemented with FBS (10%) and phytohemagglutinin for 72 h. The cells were evaluated under an inverted microscope and the mitotic spindle interrupted with the addition of colchicine (16 μg/ml). Subsequent washes and centrifugations were performed at 4°C, and the cells were fixed to slides and stained using the Wright–Giemsa staining method for karyotype assembly. Seventy different images were captured from each cell culture (*n* = 10) and at least 20 metaphases of each culture were analyzed, according to Gouveia et al. (34).

### Cell Morphology and Phenotype

The morphology and phenotype of each culture were evaluated at P10 using the same protocol. Sterile 12-well plates containing sterile circular coverslips were used. Complete culture medium (500 μl) was added to each well and 1 × 10^3^ cells were pipetted into the middle of the well for a 72-h incubation. The cell density on each coverslip was verified. When the coverslips exhibited >60% confluence, the cells were removed for morphology and phenotype analyses.

For morphology analysis, the medium was removed and the coverslips washed with DPBS three times. The cells were fixed in cold methanol (4°C) for 30 min in a refrigerator (8°C). The methanol was removed and the cells were washed three times with PBS and immersed in a 0.1% Triton-X solution for 10 min at room temperature for cell permeabilization. The permeabilizing solution was removed and the cells washed three times with DPBS and stained with hematoxylin and eosin (HE).

For immunofluorescence (IF), the medium was removed and the coverslips washed with DPBS three times. The cells were fixed with cold methanol absolute (4°C) for 30 min in a refrigerator (8°C). The methanol was removed and the cells were washed three times with PBS and immersed in a 0.1% Triton-X solution for 10 min at room temperature for cell permeabilization. The cells were blocked with a commercial solution (Protein block, Dako, Agilent Technologies) for 30 min at room temperature and primary antibodies were added to each well. We investigated pancytokeratin, cytokeratin 8/18, and vimentin expression. Information about the antibodies is provided in Supplementary Table 2. The cells were incubated with a goat anti-mouse IgG secondary antibody (Alexa Fluor 647, Life Technologies, Corporation, Carlsbad, CA, USA) and counterstained with DAPI (Sigma-Aldrich, St. Louis, MO) at a 1:10,000 dilution. As a negative reaction control, the primary antibodies tested during the procedure were omitted and replaced with a Tris buffer solution.

### Doubling Time

Cells were also evaluated for cell doubling time. The cell medium was discarded and the bottles were washed with sterile DPBS (pH 7.2) to eliminate residual FBS. For detachment of the cells from the bottle, 0.25% trypsin was added at 37°C, followed by a 5-min incubation period in a 5% CO_2_ humid atmosphere. Trypsin was inactivated with cell culture medium supplemented with 10% FBS and a 1% antibiotic/antimycotic solution. The cells were centrifuged (450 × *g*, 5 min) and the supernatant was discarded. The pellet was resuspended in 1 ml MEGM^™^ containing 10% FBS and a 1% antibiotic/antimycotic solution. The cells were then diluted in Trypan blue (Trypan blue solution, cod. T8154, Sigma-Aldrich, St. Louis, MO) in a 1:1 ratio and counted in a Neubauer chamber. For doubling time, the protocol of Caceres et al. (35) was followed. Briefly, 1 × 10^5^ cells were plated in 25-cm^5^ flasks and maintained in medium supplemented with 10% FBS and a 1% antibiotic/antimycotic solution in triplicate. Every 24 h, the cells were trypsinized and counted. This procedure was performed for 5 consecutive days to evaluate the exponential growth curve. The final number of cells for each culture was obtained via the averaging of three counts.

### Tubular Formation *in vitro*

The tubular formation assay using non-endothelial cells is an *in vitro* method to investigate the vasculogenic ability of cancer cells. The cells were cultured in three-dimensional conditions according to Salinas-Vera et al. (36). After each cell culture achieved >80% confluence, the cells were trypsinized and 50 × 10^3^ viable cells were cultured in MEGM^™^ in a 24-well plate with 250 μl of Matrigel [Matrigel^®^ Growth Factor Reduced (GFR) Basement Membrane Matrix, ^*^LDEV-Free, Corning, New York, NY, USA]. Matrigel was added to each well and air-dried for 30 min at room temperature. The medium was added and the cells were incubated in a humidified atmosphere with 5% of CO_2_ at 37°C. The cells were evaluated in an inverted microscopy every hour to determine VM formation. The experiment was performed in triplicate for each cell culture.

### Tumor Growth in Immunodeficient Mice

The ethics committee approved the experimental use of laboratory animals at Botucatu Medical School—UNESP (#1267/2018-CEUA). To evaluate cell culture tumorigenicity, 12 nude mice (BALB/c nude, C.Cg-Foxn1nu line) were acquired from the Institute of Biomedical Sciences, University of São Paulo—USP and housed in individually ventilated cages. All procedures for feeding, humidity, temperature, and light control were based on the literature (37). For *in vivo* tumorigenicity evaluation, 1 × 10^6^ cells from each cultured cell line were inoculated into different mice subcutaneously in the inguinal mammary gland region (38), and the mice were assessed once weekly for at least 60 days. After tumor growth appeared, the tumor volume was measured daily using a digital caliper. After the tumors reached 3 cm^2^, the mice were humanely euthanized and the material from the tumor was collected, formalin fixed, and paraffin embedded to confirm the malignancy via histological evaluation. Pan-cytokeratin and vimentin immunohistochemistry was performed to confirm tumor phenotype. Immunohistochemistry analysis was performed as described above using the antibodies mouse monoclonal anti-vimentin (Clone V9, Santa Cruz Biotechnology, Dallas, TX, USA) and mouse monoclonal anti-cytokeratin (Clone AE1/AE3, Santa Cruz Biotechnology) at a 1:300 dilution, overnight. The secondary antibodies, chromogen, counterstaining, and negative controls were performed as described above. The epithelial component of normal skin was used as the positive control for pancytokeratin and the dermis was used as the positive control for vimentin.

### CD31 and PAS Double Staining

The procedures for CD31/periodic acid Schiff (PAS) double staining were described by Kim et al. (39). Briefly, immunohistochemistry of the xenotransplanted tumor was performed using a rabbit polyclonal anti-CD31 primary antibody (PECAM-1, Thermo Fischer Scientific, Waltham, MA, USA) and a polymer system conjugated with peroxidase as the first stain, followed by counterstaining with 0.5% PAS and Schiff. VM is characterized by endothelial-like structures in the tumor cells that contain red blood cells stained with PAS but negative for CD31 (39). Blood vessels were identified using the CD31/PAS double staining.

### *In vitro* Migration Assay

For the evaluation of cell migration capacity, a Transwell assay was used (ThinCert^™^, Greiner Bio-One, Kremsmünster, Austria) according to manufacturer's instructions. Briefly, all cells were cultured in the same conditions described above. After reaching 80% confluence, the cells were harvested for 24 h in a medium containing 0.2% FBS. The cells were detached using 0.25% trypsin EDTA. Trypsin was inactivated with MEGM^™^ containing 5% FBS. The cells were then centrifuged (450 × *g*, 5 min) to remove the media that contained the high concentration of fetal serum and resuspended in MEGM^™^ containing 0.2% FBS. A sample (200 μl) of the solution containing each cell culture was placed on 8-μm porous membrane inserts (Greiner Bio-One) at a concentration of 1 × 10^6^ cells/ml in the upper compartment. Each insert was placed in a well of a 24-well plate that contained MEGM^™^ plus 10% FBS in the lower compartment.

Each experiment was performed in triplicate. After 24 h, the inserts were removed from the plate and placed in a new 24-well plate containing preheated trypsin. The samples were incubated in trypsin for 10 min in a humid atmosphere containing 5% CO_2_ at 37°C. Cells that were released from the bottom of the inserts were placed in a Neubauer chamber and counted according to Entschladen et al. (40).

## Results

### Cell Isolation, Molecular Phenotype of Primary Tumors, and Metastases and Cell Expansion

Ten of the 20 tumor samples grew in cell culture and were evaluated using morphology (HE) and IHC (Table 1). Six samples were classified according to Nielsen et al. (29) as HER2 overexpressing (UNESP-CM1, UNESP-CM9, UNESP-CM11, UNESP-CM60, UNESP-MM3, and UNESP-MM4), three samples were triple-negative basal-like (UNESP-CM5, UNESP-CM61, and UNESP-MM1), and one sample was triple-negative non-basal-like (UNESP-CM4).

The UNESP-CM2, UNESP-CM3, UNESP-CM6, UNESP-CM7, UNESP-CM8, UNESP-CM10, UNESP-CM12, UNESP-CM13, UNESP-CM14, and UNESP-CM15 samples did not show *in vitro* expansion. UNESP-CM2 and UNESP-CM3 cells were cultured using enzymatic dissociation overnight, but had no cellular growth after 72 h in culture conditions. UNESP-CM6 and UNESP-CM7 cultured cells were made using explants, and fungal contamination discontinued the cellular growth of both cell lines. The cells submitted enzymatic dissociation using type IV collagenase showed the best *in vitro* expansion (Table 1). UNESP-CM8, UNESP-CM10, UNESP-CM12, UNESP-CM13, UNESP-CM14, and UNESP-CM15 stopped growing and did not continue to expand to further passages.

The UNESP-CM60, UNESP-CM9, and UNESP-MM4 cultures reached more than 40 passages, and we considered these cultures immortalized. The other cell cultures were at passage 20 and still expanding.

### Contaminating Fibroblast Elimination

**Figure 1:**
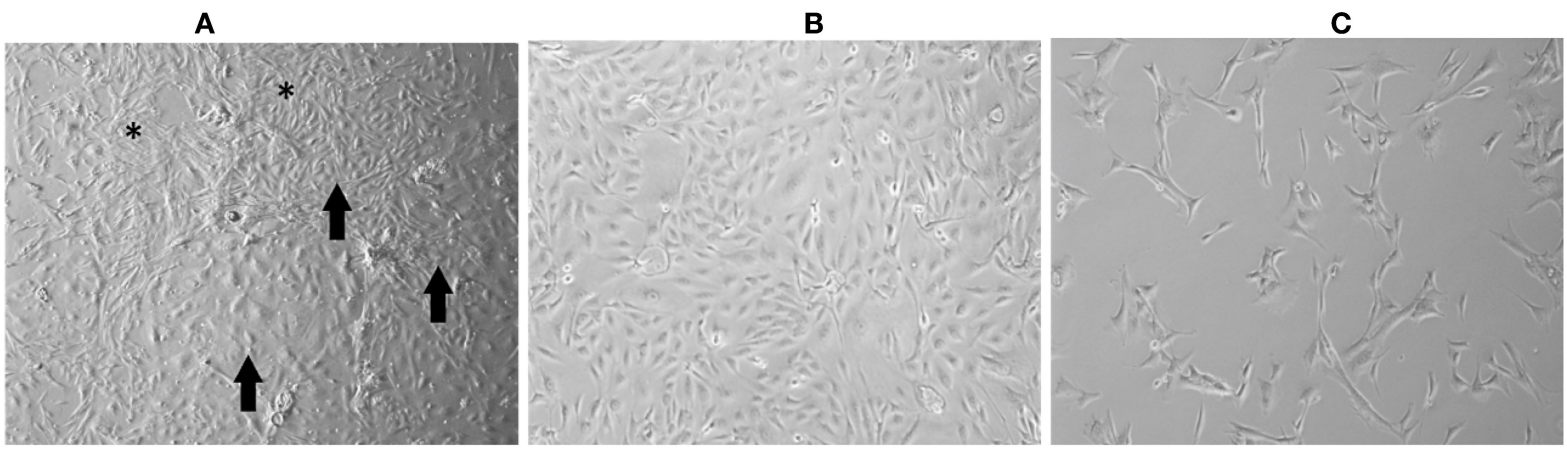
Mammary gland tumor cell culture (UNESP-CM9). **(A)** Culture of mammary gland cancer cells at passage 5 (P5). Note the heterogeneity in cell morphology, with some cells showing spindle morphology (*asterisk*) and other cell groups showing polygonal morphology (*arrows*). **(B)** Cell culture after selective trypsinization with polygonal morphology (compatible with epithelial cells) and high cell density after 48 h of culture. **(C)** Culture of cells from cold trypsinization. Note that most of the cell population has a fusiform morphology (*arrows*), and there are few cells with polygonal morphology (*arrowhead*). Note the low cell density after 30 days of cell culture.

Cell cultures prior to P5 showed a mixed morphology (spindle cells, polygonal cells, cells growing in groups, and rounded cells) (Figure 1A). After selective trypsinization at 4°C, the cells became more homogeneous and showed slower growth with spindle morphology (Figure 1B). Cells that did not show a >50% confluence after 30 days of culture were discarded. Cells subjected to trypsinization at 37°C showed a homogeneous morphology, with some cultures exhibiting a uniform spindle morphology (UNESP-CM1, UNESP-CM4, and UNESP-MM1) or polygonal morphology (UNESP-CM5, UNESP-CM9, UNESP-CM11, UNESP-CM60, UNESP-CM61, UNESP-MM3, and UNESP-MM4) (Figure 1C). The cells were grown to passage 10 (P10) and showed ~90% confluence 48 h after passage.

### Cell Karyotype

Of the 70 images captured, the best images were selected for chromosome counting. Supplementary Figure 1A shows the representations of the chromosomal alterations observed in each culture. Aneuploidies in different metaphases of the different cell cultures were identified. The UNESP-CM4 and UNESP-CM11 cultures exhibited hypoploidy of metaphase, and hyperploidy was observed in the UNESP-CM1, UNESP-CM60, and UNESP-MM3 cultures (Supplementary Figure 1B).

### Cell Morphology and Phenotype

**Figure 2:**
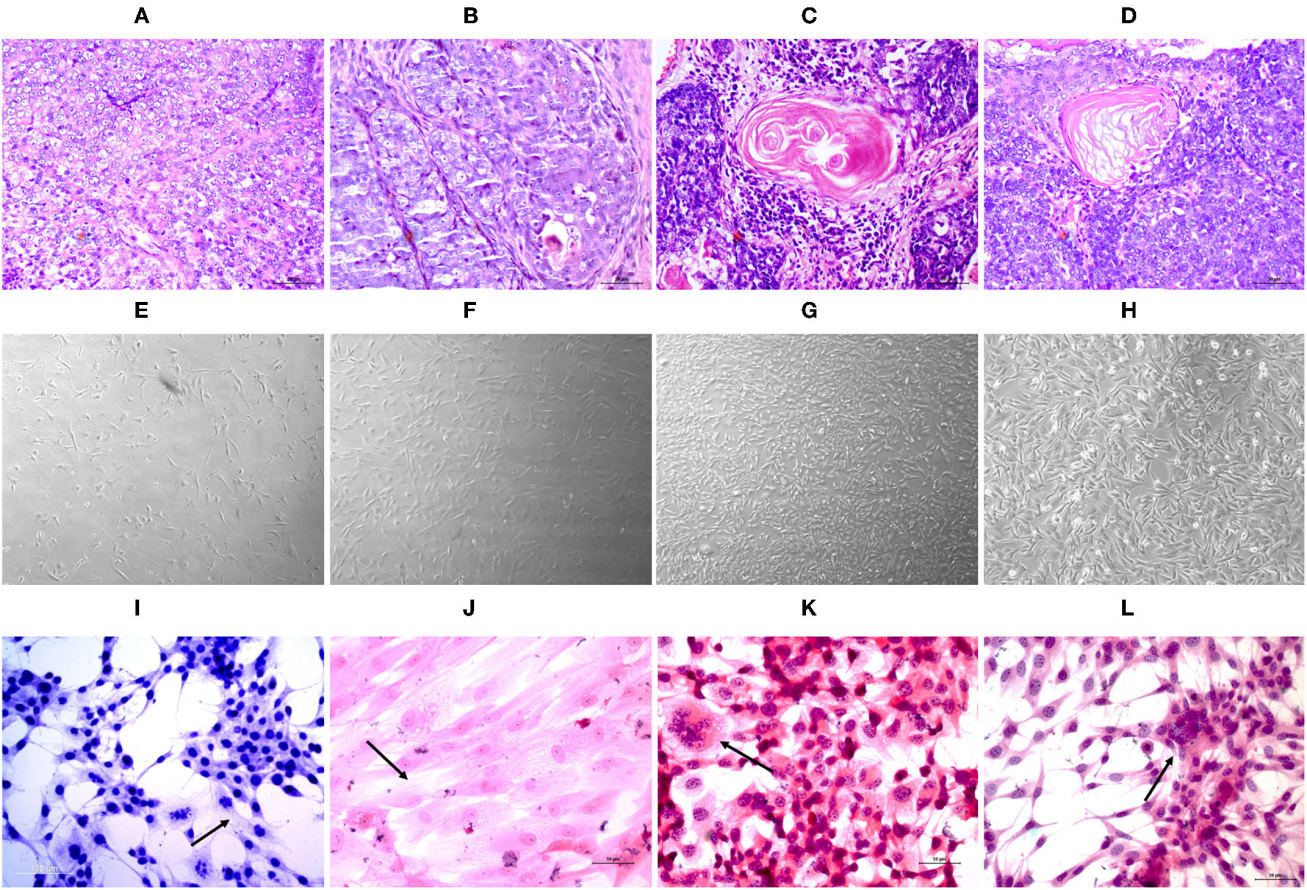
Histopathological evaluation **(A–D)** of tissue samples from canine primary and metastatic mammary tumors at ×40 magnification. UNESP-CM1 **(A)** and its bone metastasis UNESP-MM1 **(B)** and UNSP-CM60 **(C)** and its lymph node metastasis UNESP-MM4 **(D)**. *In vitro* **(E–H)** cell culture of primary **(E,G)** and metastatic **(F,H)** mammary tumor cells at ×50 magnification. HE staining **(I–L)** of cells grown *in vitro* at ×200 magnification. The *arrows* indicate multinucleated cells **(K)**, elongated cells with a large cytoplasm **(I,J)**, and colony formation **(L)**.

**Figure 3:**
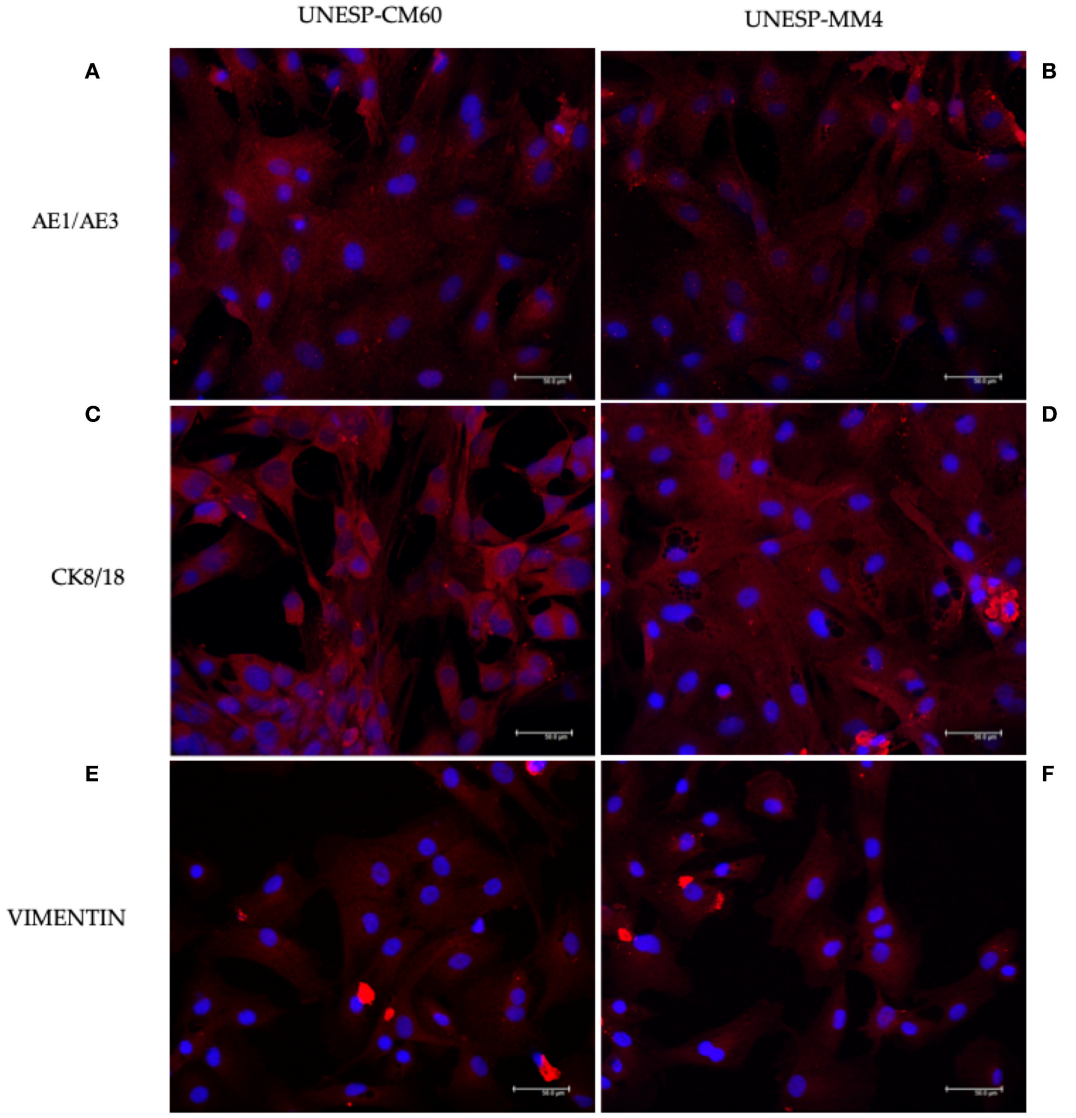
Immunofluorescence staining for pancytokeratin, CK8/18, and vimentin expressions in canine mammary gland tumor cells. The first *column* represents the cell line UNESP-CM60; the second *column* is the cell line UNESP-MM4. Pan-cytokeratin cytoplasmic expression in UNESP-CM60 **(A)** and UNESP-MM4 **(B)** cell lines. Both cell lines also expressed CK8/18 **(C,D)** and vimentin **(E,F)**.

The tumors were classified according to Goldschmidt et al. (28). Figure 2 shows the cell culture morphology, which varied from polygonal to spindle cells. Four cell lines are represented: a solid carcinoma (Figure 2A) and its bone metastases (Figure 2B) and adenosquamous carcinoma (Figure 2C) and its lymph node metastases (Figure 2D). The cells showed similar characteristics with a spindle-shaped or polygonal morphology and monolayer growth (Figures 2E–H). The morphological evaluations (Figures 2I,J) revealed that the primary and metastatic adenosquamous carcinoma tumor cells grew in monolayers in a fusiform pattern with basophilic nuclei, eosinophilic cytoplasm, and the presence of mitosis. Grade III solid carcinoma cells showed multinucleated cells and colony formation (Figures 2K,L). All the samples evaluated showed strong pancytokeratin and CK8/18 staining and were negative for p63 (Figures 3A–D). All cell cultures also showed vimentin-positive cells (Figures 3E,F). The concomitant pancytokeratin and CK8/18 expressions for all cell lines confirmed their epithelial phenotype.

### Doubling Time

The primary culture cells UNESP-CM1, UNESP-CM4, UNESP-CM5, UNESP-CM9, UNESP-CM11, UNESP-CM60, and UNESP-CM61 reached twice the number of cells initially cultured (i.e., the doubling time) at 6.31, 5.1, 7.5, 8.95, 7.45, 11.79, and 13.06 h, respectively. The UNESP-MM1, UNESP-MM3, and UNESP-MM4 metastasis cultures exhibited doubling times at 25.41, 34.17, and 10.28 h, respectively.

### Vasculogenic Mimicry *in vitro* and *in vivo*

**Figure 4:**
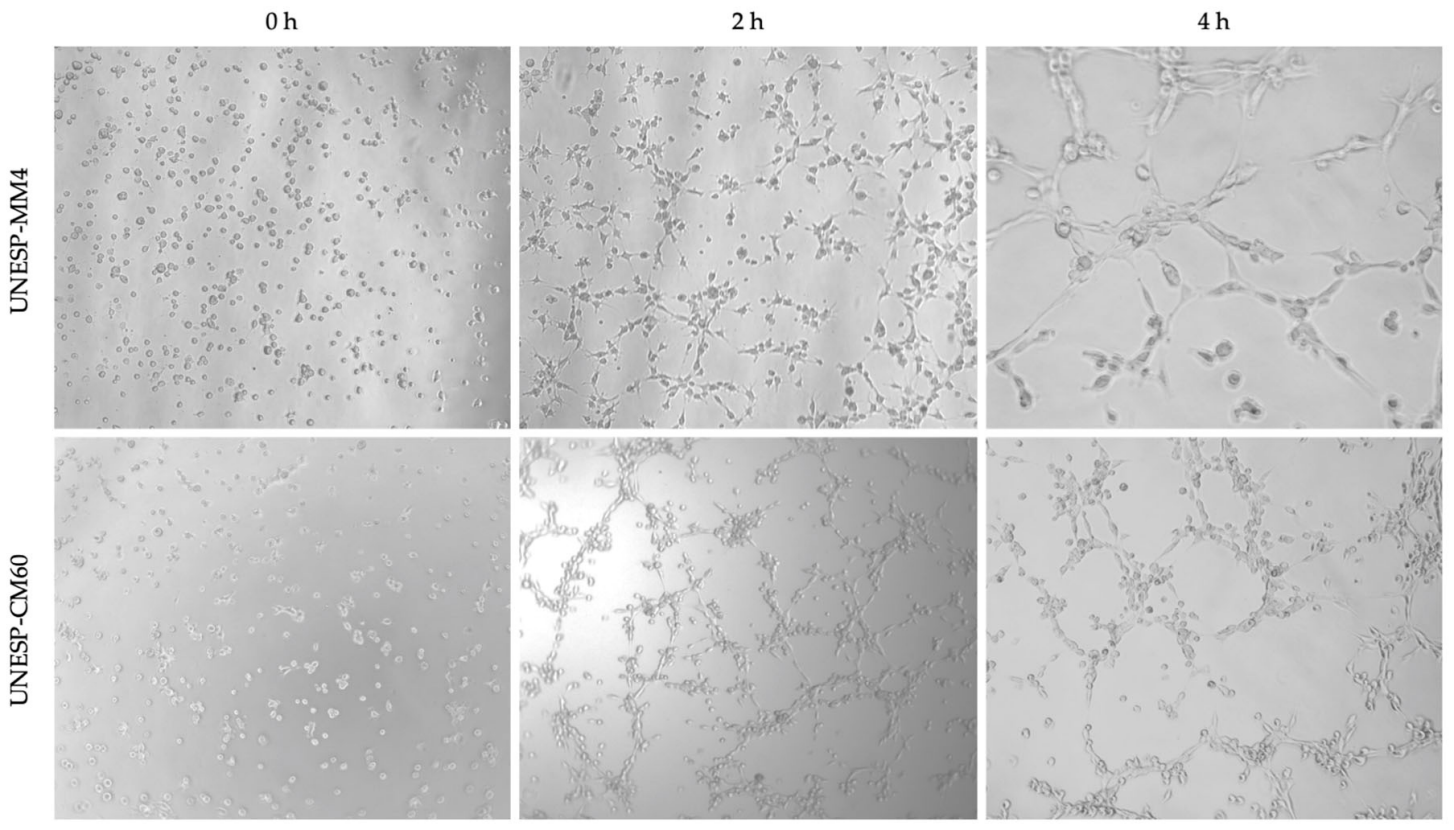
Three-dimensional experiment to evaluate the vasculogenic mimicry ability *in vitro* of cells from a primary carcinoma tissue (UNESP-CM60) and its metastasis (UNESP-MM4). Three different moments are observed in both cell cultures. Complete tubular formation occurred at 4 h for the two cell lines (×50 magnification).

**Figure 5:**
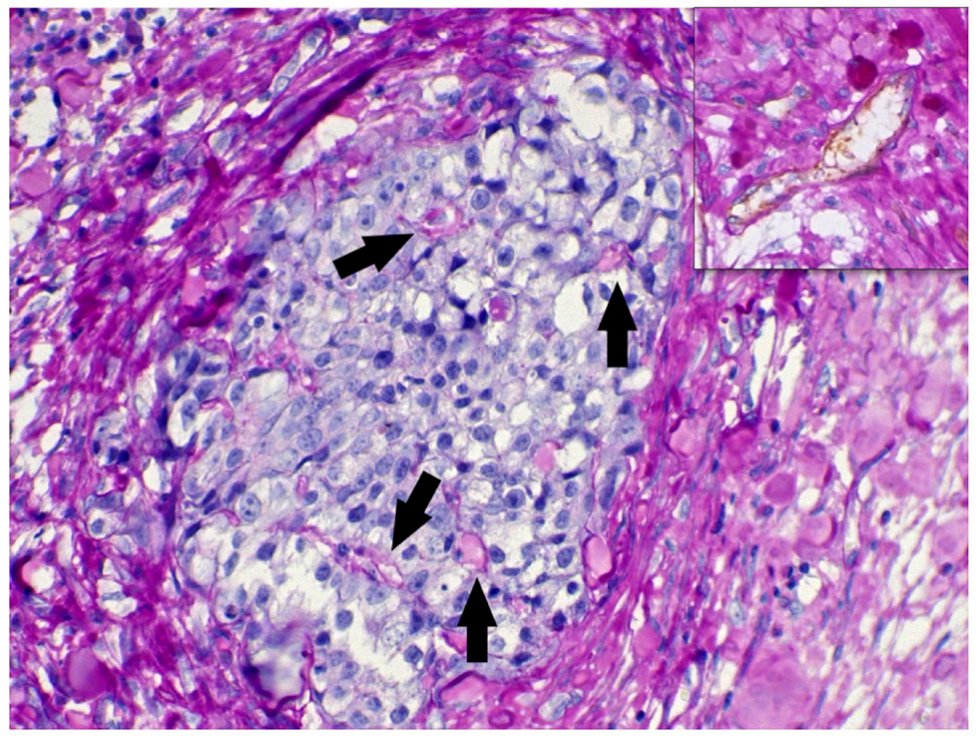
CD31/periodic acid Schiff (PAS) double staining of the xenotransplant tumor. A group of neoplastic cells in a solid formation is shown. The *arrows* indicate PAS-positive endothelial-like structures formed by neoplastic cells. The *highlighted area in the top* shows a blood vessel double stained for CD31/PAS.

**Figure 6:**
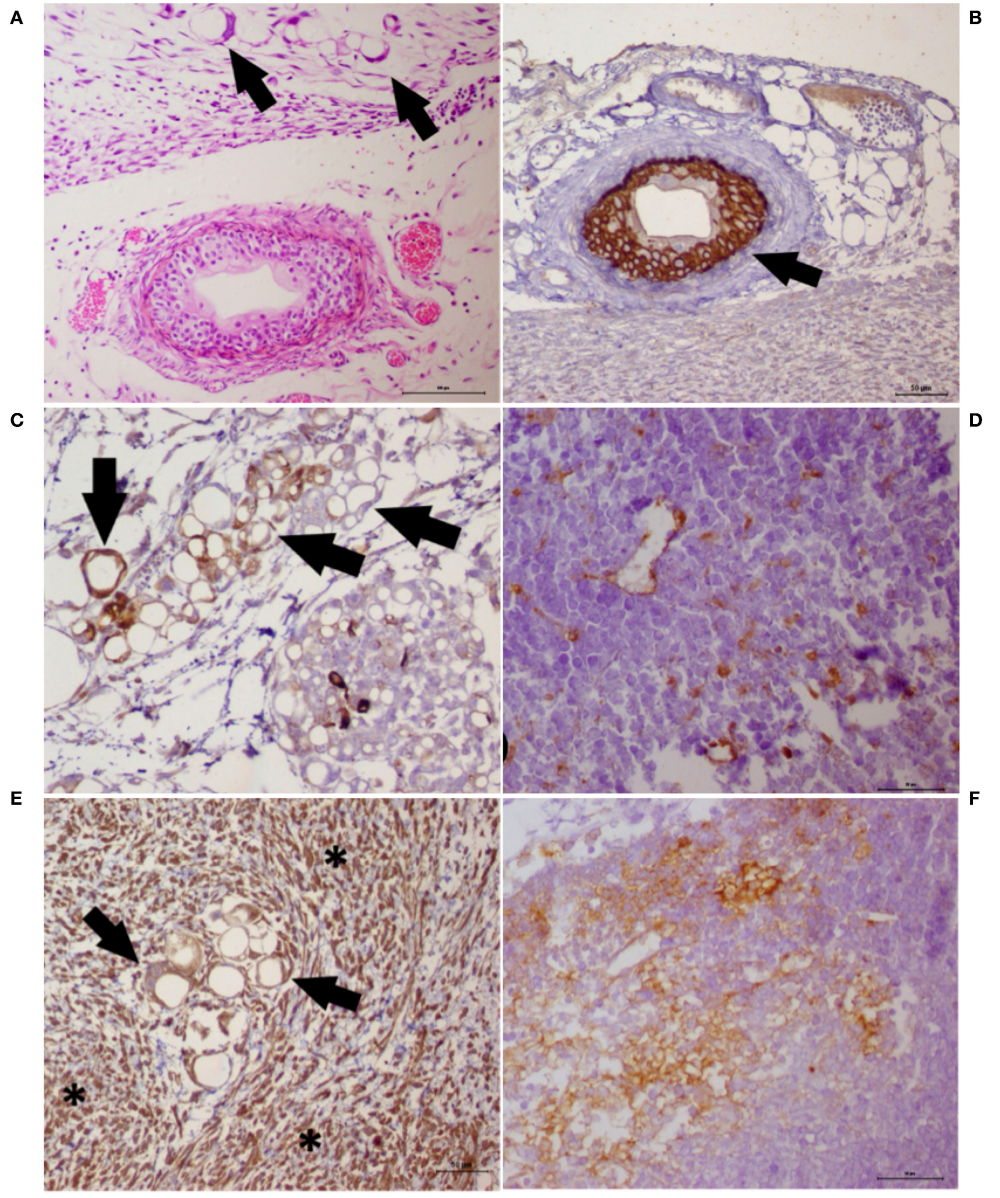
Histochemistry and immunohistochemistry analyses of a tumor growth (xenograft) from the cancer cell lines UNESP-MM4 and UNESP-CM60. **(A)** HE staining showing neoplastic cells forming vascular-like structures (*arrows*) and the presence of a blood vessel showing tumor cells growing into the blood vessel from the UNESP-MM4 cell line. **(B)** Positive pancytokeratin expression in the neoplastic cells growing inside a blood vessel (*arrow*) from the UNESP-MM4 cell line. **(C)** Positive cytokeratin 8/18 expression of neoplastic cells forming vascular-like structures and confirming its epithelial origin. **(D)** Vascular-like structure from the xenotransplantation of the UNESP-CM60 cell line. Note the scattered cytokeratin 8/18 expression of neoplastic cells, including vascular-like structures, which confirms its epithelial origin. **(E)** Vimentin expression of tumor growth from the UNESP-MM4 cell line. Note the positive vimentin expression in neoplastic (*arrows*) and stromal (*asterisk*) cells. **(F)** Vimentin-positive expression in tumor growth from xenotransplantation of the UNESP-CM60 cell line.

*In vitro* tubular formation was identified in four of the 10 cell cultures. The cell lines UNESP-CM1, UNESP-CM9, UNESP-CM60, and UNESP-MM4 exhibited *in vitro* VM formation from 4 h (Figure 4) to 6 h, and vasculogenic mimicry-like structures were disrupted after 6 h.

One primary cell culture (UNESP-CM60) and its respective metastasis (UNESP-MM4) showed *in vivo* tumorigenicity (two of the 10 cell cultures) (Supplementary Figure 2). Macroscopic growth was evident 50 days after cell administration in nude mice (BALB/c nude, C.Cg-Foxn1nu line), and histology revealed a tumor with high VM formation. Vasculogenic mimicry was characterized by neoplastic cells forming PAS-positive tubules containing plasma and red blood cells (Figure 5). VA was also observed in the internal positive controls, which contained PAS-positive blood vessels associated with CD31-positive endothelial cells (Figure 5). Both cell lines were neoplastic cells with evident nucleoli that formed endothelial-like structures mimicking capillaries (Figures 6A–E). These capillary-like structures were positive for pancytokeratin and vimentin (Figures 6B–F). Notably, the metastatic cell line UNESP-MM4 also showed intravascular growth (Figure 6A). Several blood vessels were observed in the tumor periphery with the intravascular growth of pancytokeratin-positive cancer cells (Figure 6B).

### *In vitro* Migration Assay

**Figure 7:**
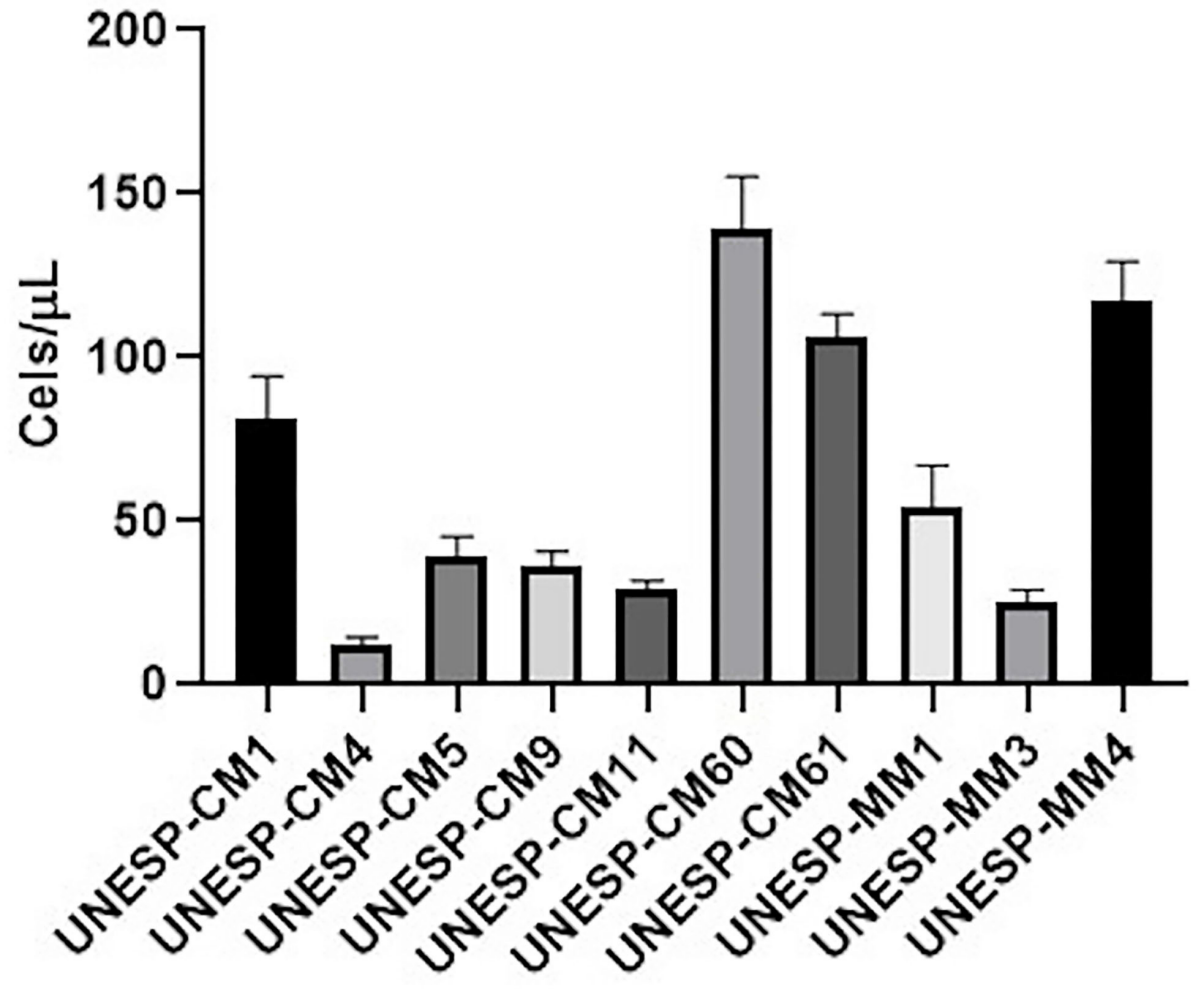
Cell invasion of all cell lines using the Transwell assay. The migration ability in all cell lines is shown. The metastatic cell lines exhibited a higher migration ability.

To evaluate the invasion capacity of all cell lines, the Transwell assay was performed to measure cellular migration. The numbers of migrating cells for UNESP-CM1, UNESP CM4, UNESP-CM5, UNESP-CM9, UNESP-CM11, UNESP-CM60, UNESP-CM61, UNESP-MM1, UNESP-MM3, and UNESP-MM4 were 80 ± 12, 11 ± 2, 38 ± 5, 36 ± 4, 29 ± 2, 147 ± 15, 107 ± 7, 54 ± 13, 27 ± 3, and 113 ± 12, respectively (Figure 7).

## Discussion

We cultured and characterized seven primary cell cultures from mammary gland tumors and three cultures from metastases *in vitro* and *in vivo* (xenotransplant animals). The techniques used were adequate to establish the cell lines. There is a need for *in vitro* and natural models for studies in veterinary and comparative oncology. We also found four cell lines that exhibited VM, which is a feature of aggressive mammary tumors.

Canine mammary gland tumors are one of the most important tumors in intact female dogs and may be considered a model for studying human disease. Several cell lines are used as a preclinical model for understanding BC development and progression and for the investigation of the antitumor effects of new drugs (41–44). Compared to human BC, there are fewer canine mammary gland tumor cell lines to study (37, 45–47). Mammary tumors of dogs are classified by the receptors expressed as luminal A, luminal B, triple-negative basal-like, triple-negative non-basal-like, and overexpressing HER2, which are established in humans. Triple-negative tumors do not express ERα, PR, or HER2 receptors and may be divided into basal-like and non-basal-like. Basal-like tumors express EGFR and/or CK5/6, and non-basal-like tumors are EGFR and/or CK5/6-negative (48). Human basal-like triple-negative tumors have the worst prognosis and do not have a defined efficient therapy (10, 29, 48). Luminal tumors are assessed by the presence of hormone receptors (ERα and PR), Ki-67, CK5/6, and EGFR. Ki-67 is a cell proliferation marker, and these tumors are divided into two subtypes: luminal tumor A if the amount of Ki-67 is low and luminal tumor B if the amount of Ki-67 is high. CK5/6 and EGFR help in the identification of basal-type tumors with aggressive behavior (48). The establishment of HER2-overexpressing tumors in dogs is challenging because HER2 overexpression is generally associated with the absence of HER2 genomic amplification (49, 50). HER2 alterations in normal mammary cells were demonstrated previously using chromogenic *in situ* hybridization (CISH) (50). Because an increased number of HER2 copies was identified in normal mammary gland tumors using CISH, its role in canine tumor development is not certain. We established HER2-overexpressing cells (UNESP-CM1, UNESP-CM9, UNESP-CM11, UNESP-CM60, UNESP-MM3, and UNESP-MM4), which may be used in future comparative models to understand the role of the HER2 protein in canine mammary gland tumors.

To establish the primary cell cultures, we used different methodologies to identify the most effective protocol for isolating neoplastic epithelial mammary cells. The cultures obtained from the explant samples presented several problems in their establishment, which were primarily related to contamination during cultivation. Therefore, we used protocols based on the use of enzymatic dissociation. We used collagenase types I and II for different incubation times (3, 4, 12, 24, and 48 h), with no success (data not shown), but 0.05% type IV collagenase showed better results. However, incubations of 0.05% type IV collagenase for 24 and 48 h induced cell damage and death, with no cell growth (data not shown). Samples incubated with 0.05% type IV collagenase for 4 h showed no cell damage and satisfactory culture expansion. Therefore, we standardized the incubation time of up to 4 h for enzymatic dissociation.

After the different cell cultures were established, the cells exhibited a heterogeneous morphology. Therefore, selective trypsinization of cells was performed to eliminate fibroblasts and stimulate cell clone formation (51). After the initial expansion, the cells were used for characterization at passage 10 (P10) because cells from lower passages tended to have a heterogeneous morphology and cells in higher passages exhibited a greater number of chromosomal alterations related to cell culture conditions (52, 53). We did not find contaminating fibroblasts in the cultures after P9. We used two different strategies to eliminate fibroblasts: a culture medium that contained specific epithelial cell growth factors and selective trypsinization. Both strategies effectively eliminated fibroblasts and selected epithelial cells during cell expansion, which was confirmed by the expression of epithelial markers by neoplastic cells.

Some studies showed different cell line stabilities in cell phenotype and functional characteristics from P10 up to P30, regarding (54, 55) and genomic stability from P5 to P13 (52, 53, 56). We used cells in a lower passage based on a previous literature that described genomic stability in lower passages and the use of all cells in a similar passage for experimental homogeneity. However, it is important to grow cell lines until higher passages (between P40 and 60) and evaluate tumor stability prior to the commercial use of these cells. The cell lines exhibited a monolayer growth and similar size and morphology. The cells showed a spindle shape, a high nucleus-to-cytoplasm ratio, and tight cell–cell adhesion. The high ratio of the nucleus and cytoplasm size is generally associated with malignant tumor behavior (57). HE staining revealed basophilic nuclei and an eosinophilic cytoplasm with the presence of mitosis, and some cells appeared multinucleated with colony formation. Our cell lines also expressed vimentin, which was reported in MCF7 and HeLa cells cultured *in vitro* (58, 59). Cells in culture conditions must change their cytoskeleton for flask attachment, and it is common to observe vimentin expression in epithelial cells (60). This expression was previous explained by posttranslational modification during cell culture conditions (35). The expression of cytokeratins and vimentin in BC is related to malignancy (61). Although we used markers and experiments from the literature (17, 20, 62), other markers may be used to confirm the tubular-like structures as VM. One limitation of our study was the absence of investigation of further markers related to VM, such as VE-cadherin, transforming growth factor-β1 (TGF-β1), and EpCam (14, 17, 19, 63). The evaluation of these markers in future research may be valuable for a better characterization of VM markers in canine mammary gland tumors.

The doubling times of each culture were analyzed. The shortest time occurred in UNESP-CM4 (5.1 h) cells, and the longest time occurred in UNESP-MM3 (34.17 h) cells at passage 10, which shows rapid growth in all cell lines. These results are similar to those of Cordeiro et al. (37) in which the doubling times of two different cells lines from canine mammary gland tumors cultured *in vitro* were 26 and 42 h, and one cell line was more malignant than the other based on the invasion potential and *in vivo* tumorigenicity. The migration ability of each cell line was assessed as a tumorigenic characteristic, and all cell lines migrated in the Transwell model to different degrees. Cell lines from more aggressive histological subtypes, such as solid and adenosquamous tumors, showed a higher migration rate. These results demonstrate a direct relationship of the primary tumor histological subtype with the migration ability *in vitro*.

The characterized cells were extracted from tumors that expressed HER2 or were triple-negative. Therefore, these cells may be used as a model for breast tumors in women that express HER2 and triple-negative tumors, which are the most aggressive subtype with a worse prognosis and fewer therapeutic possibilities (48). The UNESP-CM60 and UNESP-MM4 cells were generated from a primary tumor and its metastasis. The primary tumor and metastasis exhibited an adenosquamous histological pattern. There are no cell lines with these characteristics in the veterinary literature, and both cells represent a unique opportunity to study the metastatic phenotype. The UNESP-CM60 culture and its lymph node metastasis (UNESP-MM4) were HER2-positive, which provides the opportunity to study this phenotype in dogs and compare it to human BC.

An interesting finding was the *in vitro* VM in four different cell lines. Tumor cells that exhibit VM are highly malignant and capable of penetrating the endothelium for tumor invasion and metastasis (16). Two of our cell lines also showed VM ability *in vivo* (tumor from xenotransplant). VM formation is associated with aggressive melanoma cells, but not non-aggressive cells (14), and high tumor grade, invasion and metastasis, and poor clinical prognosis in hepatocellular carcinomas (15). VM is commonly described in human and canine inflammatory mammary tumors, and it is likely related to tumor aggressiveness and metastasis capacity (24, 25). The VM results are consistent with the results from the tumorigenicity assay in which two of the cellular types that were capable of VM formation produced tumors *in vivo*. HE revealed that the tumors from the xenotransplants had high VM formation, which shows the aggressiveness of this neoplasia *in vivo* and *in vitro*. The ability to form VM *in vitro* and *in vivo* supports the use of these cells as a preclinical model for canine mammary gland tumors.

Four of our 10 tumor cell lines showed tumorigenicity *in vivo*. This result is similar to that of Cordeiro et al. (37), who showed that only one in two different cell lines grew *in vivo*. Although the cells exhibited epithelial characteristics, such as morphology, growth pattern, phenotype, protein expression, and tumorsphere formation, the tumor did not grow *in vivo* (37). Tumor growth *in vivo* depends on many factors, such as the administration location, cell concentration, tumor heterogeneity, and the immune system of the animal (64). Our results may be explained by the animal model used in the study. BALB-c nude mice are immunodeficient because they lack a thymus and do not produce T cells. However, this mouse model produces other immune cells, such as B cells and natural killer cells, that affect tumor growth *in vivo*. Histological evaluations of xenograft tumors revealed a high inflammatory infiltrate in all tumors (*n* = 4), which indicates that other immune cells infiltrated the tumors. Nude mice with no B cells or natural killer cells may accurately evaluate *in vivo* tumorigenicity (65). However, the use of more highly immunocompromised mice requires the use of a pathogen-free laboratory system. Unfortunately, our institution does not have the necessary infrastructure to accommodate these immunocompromised mice. Therefore, a major limitation of our study is the lack of structure to evaluate cell tumorigenicity in a less immunogenic mouse.

The cell invasion capacity in Transwell assays showed a higher migration of UNESP-CM60, UNESP-CM61, and UNESP-MM4 cells. These results support the *in vivo* tumorigenicity and invasion ability of UNESP-CM60 and UNESP-MM4 cells and confirm the malignant behavior and characteristics of these lines.

Some studies showed that drug testing and behavioral studies of tumors were performed efficiently using mammary tumor cells cultured *in vitro* (35, 66, 67). The establishment and characterization of new cell lines is significant and provides a useful cell model for studies of basic tumor biology, development, and other uses (35, 66). There is no consensus on the importance of HER2 in mammary gland cancer in veterinary medicine (68). However, an increase in HER2 expression in dogs was related to cell pleomorphism and the number of mitosis figures, and this relationship was observed in humans (69, 70). Triple-negative cells are more important due to their worse prognosis, and these cells will assist in the understanding and study of these types of tumors in dogs and humans (4, 67).

Overall, our study established new canine mammary gland tumor cell lines to increase our understanding of this disease in dogs. Several researchers characterized and established canine mammary tumor cell lines that showed tumorigenic potential, but only one study confirmed VM formation in canine inflammatory mammary tumor cells (25). We provided valuable information on cell lines that formed VM *in vitro* and *in vivo* and exhibited *in vivo* tumorigenicity. VM is a potential prognostic and predictive marker in tumors, but it is difficult to find *in vivo* models to understand this phenomenon (71). Our study provides two canine mammary gland tumor cells with the ability to form VM *in vivo* as a unique model for understanding this phenomenon.

## Conclusions

In summary, we established and characterized 10 cell lines and xenografts from canine mammary gland carcinomas and metastases. The cells cultured *in vitro* demonstrated morphological and phenotypic similarities, but had tumorigenicity differences. Four cell lines exhibited VM ability *in vitro*, and two of these cell lines showed *in vivo* tumorigenicity related to malignancy and aggressiveness. Therefore, the described cell lines may be used in the future for clinical investigations, therapeutic targets, and for studying gene targets and pathways.

## Data Availability Statement

The raw data supporting the conclusions of this article will be made available by the authors, without undue reservation.

## Ethics Statement

The animal study was reviewed and approved by Ethics Committee on Animal Use (CEUA) of Veterinary Teaching Hospital of São Paulo State University.

## Author Contributions

PF, AB, AL, MP, PK, RL-A, and CF-A conceptualized the study. PF, AB, AL, MP, PK, and CF-A collected the data. PF, AL, and CF-A contributed to the writing—original draft preparation. PF, AL, RL-A, and CF-A did the writing—review and editing. CF-A supervised the study. All authors read and agreed to the published version of the manuscript.

## Conflict of Interest

The authors declare that the research was conducted in the absence of any commercial or financial relationships that could be construed as a potential conflict of interest.
