# Supplementary material for: Establishment and Characterization of Canine Mammary Gland Carcinoma Cell Lines With Vasculogenic Mimicry Ability in vitro and in vivo
Source: Front Vet Sci. 2020 Oct 27;7:583874. doi: 10.3389/fvets.2020.583874 (PMC7655132; doi:10.3389/fvets.2020.583874)
Supplement: Supplementary file 1 [file Data_Sheet_1.PDF]

Supplementary Table A1. Primary antibodies used in immunochemistry to characterize the molecular phenotype from mammary gland tumors.

Antibody	Manufacture	Clone	Dilution	Imunolocalization
P63	Dako, Agilent Technologies, Santa Clara, CA, USA	4A4	1:100	Nuclei
HER2	Roche Diagnostics, Risch-Rotkreuz, Switzerland	4B5	1:400	Membrane
ERα	Santa Cruz Biotechnology®, Santa Cruz, CA, USA	C311	1:50	Nuclei
PR	Roche Diagnostics, Risch-Rotkreuz, Switzerland	1E2	Prediluted	Nuclei
Ki-67	Dako, Agilent Technologies, Santa Clara, CA, USA	MIB1	1:50	Nuclei
CK5/6	Dako, Agilent Technologies, Santa Clara, CA, USA	D5/16B4	1:10	Citoplasm
EGFR	Invitrogen, Thermo Fisher Scientific Corporation, Carlsbad, CA, USA	31G7	1:20	Citoplasm

Supplementary Table A2. Primary antibodies used in cell immunofluorescence to characterize the cell clone (cell origin) that was expanded in each culture.

Antibody	Manufacture	Clone	Dilution	Imunolocalization
Pan-citoqueratin	Invitrogen, Thermo Fisher Scientific Corporation, Carlsbad, CA, USA	AE1/AE3	1:300	Citoplasm
Vimentin	Invitrogen, Thermo Fisher Scientific Corporation, Carlsbad, CA, USA	V9	1:300	Citoplasm
CK8/18	Novocastra, Vision BioSystems Ltd, Newcastle, UK, Europe	5D3	1:600	Citoplasm

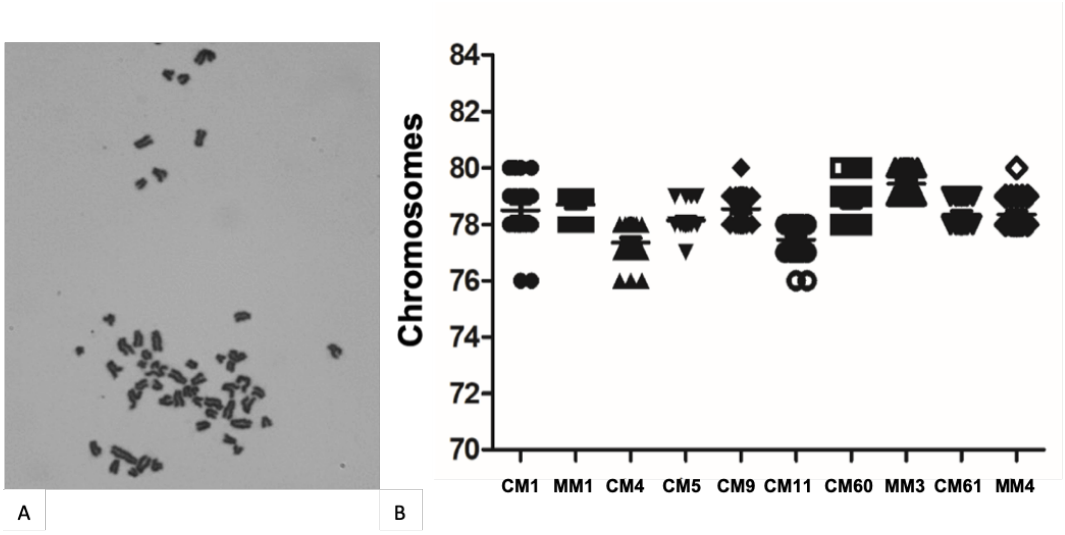

Supplementary Figure A1. Chromosomal preparation from cell culture of female dog mammary gland carcinoma (A). Graphic representation of chromosomal alterations in different cultures of canine mammary gland cancer and its metastases (B).

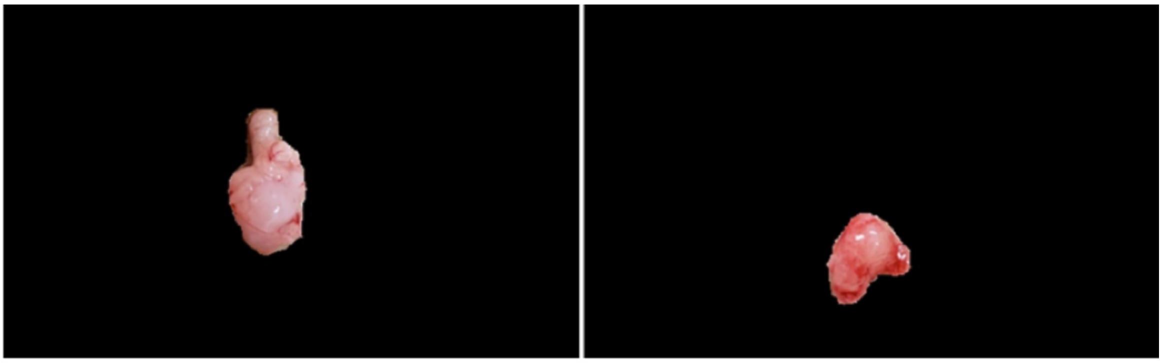

A B

Supplementary Figure A2. Macroscopic appearance of tumours after 60 days of growth. A: Tumour growth from UNESP-MM4 cell line. B: Tumour growth from UNESP-CM60 cell line.
